# Current state of the art in laparoscopic colorectal surgery for cancer: Update on the multi-centric international trials

**DOI:** 10.1186/1750-1164-6-5

**Published:** 2012-07-30

**Authors:** Jennifer K Lee, Conor P Delaney, Jeremy M Lipman

**Affiliations:** 1 Department of Surgery, University Hospitals, Case Medical Center, Case Western Reserve University, Cleveland, OH, USA; 2 Department of Surgery, MetroHealth Medical Center, Case Western Reserve University, Cleveland, OH, USA

**Keywords:** Laparoscopy, Colorectal cancer, Cost, Classic, Color

## Abstract

Laparoscopic colectomy is now widely applied to cases of malignancy, supported by early data from several large randomized controlled trials. Long-term follow-up is now available from those trials, supporting equivalency of cancer-free and overall survival for open and laparoscopic resections. This promising data has inspired further exploration of other applications of laparoscopic techniques, including use of single incision laparoscopy. This article reviews recent reports of long-term data for colorectal cancer resection from four randomized, prospective international trials.

## Introduction

Colorectal cancer remains the second leading cause of cancer-related death in Western countries [1]. Despite advancements of medical treatments, surgical excision remains the only definitive therapy for colorectal cancer. The first reported laparoscopic sigmoid resection in 1991 inspired the application of laparoscopy to a range of colorectal disease, including cancer [2]. The reported short-term benefits of laparoscopy include less postoperative pain, faster return of bowel function, shorter hospitalization, and overall improved quality of life [3]. In spite of these benefits, reports of port-site tumor recurrence and the ability to perform adequate lymphadenectomy raised concerns for the safety and adequacy of laparoscopic oncologic resections [4]. These concerns prompted the design of several studies, including four large randomized controlled trials (RCT) that are now generating 3 and 5-year follow-up data. Short-term and 3-year outcomes from three studies supported that overall and cancer-free survivals with laparoscopy were not significantly different from conventional open resection.

The objective of this review is to provide an update on the long-term outcomes from four large RCTs. The field of laparoscopy continues to evolve with newer techniques and instruments, providing both better outcomes and a wider range of application in the field of colorectal surgery. This article also aims to review the available outcomes regarding laparoscopic rectal cancer resection, as well as utilization of the single-site incision laparoscopic (SIL) technique.

## Colon cancer

### COST: clinical outcomes of surgical therapy study group

COST analyzed 863 patients among 48 centers in the United States and Canada. With insufficient data suggesting superiority of laparoscopy to open approach, the authors designed COST as a non-inferiority trial. Data was accrued from 1995 through 2001. It excluded cancers located in the transverse colon and rectum. The primary endpoint was time from randomization to tumor recurrence [5].

Early data was published in 2002, outlining the benefits of laparoscopy in the early postoperative period. Patients reported improved quality of life and a significantly shorter hospital stay compared to the open group (5 vs. 6 days, p < 0.001). The laparoscopic group also required one less day of analgesia. These benefits have been supported by the other RCTs [2]. The conversion rate to open resection was 21%, and remained consistent throughout the study course. Three-year data showed no difference in overall survival (lap: 86%, open: 85%; p = 0.51) or recurrence (lap: 16%, open: 19%; p = 0.32) [5].

Five-year data was available on 90% of patients and published in 2007. COST showed that with 170 recurrences and 252 deaths, cancer-free and overall 5-year survival was similar between open and laparoscopic groups. The rate of recurrence, along with the sites of those recurrences, was also similar (lap: 76%, open: 74%; p = 0.93). Although not adequately powered, an exploratory subset analysis was done to evaluate whether conversion to open operation impacted the outcome measures. This analysis did not identify a significant difference between groups for either cancer-free survival or recurrence [6].

### Color: colon cancer laparoscopic or open resection

COLOR included 29 European centers with data from 1076 patients accrued from 1997 through 2003. As in the COST trial, transverse colon and extra-peritoneal rectal cancer were excluded. COLOR, however, also excluded those with a body mass index greater than 30 as obesity was considered a technical challenge at the time of this study’s design. The primary endpoint was cancer-free survival at 3 years. Similar to the COST study, COLOR was designed as a non-inferiority RCT. Also like COST, COLOR included surgical teams that had performed at least 20 laparoscopic colectomies, confirmed by evaluation of videotaped operations. The rate of conversion was 17% mostly (34%) due to encountering unexpected bulky disease. The majority of patients in this trial had only barium enema radiography and colonoscopy pre-operatively. Only 5% of the patients in this trial had pre-operative computerized axial tomography (CT) evaluation. This emphasizes the importance of preoperative radiographic staging with imaging tools such as magnetic resonance imaging (MRI) and CT to facilitate operative planning.

Short-term results were published in 2005, describing laparoscopy as a longer operation (lap: 145 min, open: 115 min; p < 0.0001) with less blood loss (lap: 100 cc, open: 175 cc; p < 0.0001). There were no significant differences in oncologic outcomes including the rate of positive margins (p = 1.0) or in the number of lymph nodes harvested (p = 0.35). Postoperative morbidity was also similar for both groups with no significant differences in postoperative pulmonary or cardiac events, anastomotic failures or wound infections [7].

At 3 years, COLOR found recurrences, whether local, distant or combined, were similar between both groups. Overall and cancer-free survival were not significantly different, regardless of disease stage. The 3-year cancer-free survival for all stages was 72.4% in the laparoscopic group and 76.4% in the open group (p = 0.7). Overall survival at 3 years for all stages was 81.8% in the laparoscopic group and 84.2% in the open group (p = 0.45) [8].

Although the actual difference in cancer free survival at 3 and 5 years was small, the study did not reach its predetermined non-inferiority margin and thus could not rule out a difference in disease-free survival at 3 years in favor of open colectomy. The authors selected 7% as the cutoff to show non-inferiority regarding the difference in cancer-free survival (survival in patients having laparoscopic operation subtracted from that of those having open operation). At 3 years this difference was 2.0% (95% CI −3.2 to 7.2) and at 5 years was 1.4% (−4.6 to 7.5). Because the upper limit of the 95% confidence interval is greater than 7%, non-inferiority could not be confirmed. This difference is very small, however, and the clinical implications are unlikely to be significant. Additionally, if patients are analyzed by the treatment they actually received rather than by intention to treat, this difference does meet their criteria for non-inferiority (1.7% 95% CI–3.5 to 6.9). Also, 3 and 5 year differences in overall survival meet the criteria for non-inferiority (3 years 2.4% (95% CI −2.1 to 7.0) and 5 years 0.4% (−5.3% to 6.1), though this was not a primary endpoint of the study.

### Classic: conventional versus laparoscopic-assisted surgery in colorectal cancer

CLASICC included 794 patients accrued from 27 United Kingdom centers between 1996 and 2002. The CLASICC study stood out among the other RCTs during this time by including patients with rectal cancer. Patients in this study were randomized in a 2:1 basis, such that 526 were in the laparoscopic group and 268 in the open group. Of the 794 patients, 413 (52%) had colon cancer. The findings for patients with rectal cancer will be discussed separately. The short-term primary endpoints were rates of positive circumferential and longitudinal resection margins, proportion of Dukes’ C2 tumors and in-hospital mortality. Long-term endpoints were defined as survival, recurrence and quality of life at 3 and 5 years, of which results are now available.

Short-term results were reported in 2005. Overall, there were no significant differences noted with regard to the stated short-term endpoints and quality of life. There was no significant difference in the number of lymph nodes or the number of positive margins for colon cancer, supporting that laparoscopy provided an adequate oncologic resection. The group suggested that this would predict a local recurrence rate no higher and a cancer-related survival no shorter than open resection [9]. These favorable early results of CLASICC prompted a 2006 modification of the National Institute of Health and Clinical Excellence guideline to consider laparoscopic resection as an option for those with colon cancer. These favorable findings from early results echoed those of the other international RCTs for colon cancer.

The CLASICC group did find more complications among patients converted to open operation. The rate of conversion for colon cancer was 25% and for rectal cancer was 34%. The most common cause for conversion in both groups was fixation of the tumor. Of rectal cancers, 20% were converted because the tumor was deemed inaccessible laparoscopically. This may attest to the greater technical challenge of laparoscopy for low anterior resection (LAR) and abdominoperineal resections (APR). A learning curve was identified with a decrease in overall rate of conversions to 16% by year 6 from 38% noted in year 1. Converted cases had a significantly higher rate of intraoperative complications, including intraoperative hemorrhage or arrhythmia (p = 0.002). Converted cases also had a trend towards higher death rate than open or laparoscopic arms (9% vs. 5% and 1%, respectively; p = 0.34), though this was not statistically significant. The main cause of death in these patients was cardiorespiratory failure. These effects on overall survival persisted at 5 years, with lower survival for those who were converted (p = 0.033). There was no significant difference in distant recurrence rates for converted cases [9,10].

Recently published 5-year data supports the initial short-term and 3-year results, strengthening the argument that laparoscopy provides short-term benefits that do not compromise long-term outcomes. Overall survival for both laparoscopic and open resections was similar (lap: 57.9%, open: 58.1%; p = 0.848). Regarding colon cancer, the overall survival was again not significantly different between treatment arms (lap: 55.7%, open: 62.7%, p = 0.253). [10] Similarly, there was no difference in 5-year cancer-free survival for colon cancer (lap: 57.6%, open: 64.0%, p = 0.399). In total, one wound site recurrence was recorded in the open arm and nine in the laparoscopic arm. There were no additional port-site recurrences reported at 3 or 5 years [10,11].

### Barcelona study

A single-center RCT included data from 219 patients with colon cancer greater than 15 cm from the anal verge accrued from 1993 through 1998. A single surgical team performed all procedures. The primary endpoint was cancer-related survival with data analyzed according to the intention-to-treat principle. Short-term data suggested that those in the laparoscopic group recovered faster and had lower morbidity. Median follow up was 98 months. Long-term results were published in 2008. Although overall survival and rate of recurrence favored the laparoscopic group, this was not statistically significant.

An interesting finding in this study was a higher probability of cancer-related survival in the laparoscopic group, specifically those with locally advanced disease. These results have not been reproduced by other trials, however. The group postulates that the surgical stress response is less in laparoscopy and therefore may play a role in the reported oncologic advantage. The underlining mechanism is attributed to less surgical trauma, leading to less cytokine release and thus less tumor stimulation. The role of vascular endothelial growth factor in angiogenesis and tumor growth has been of particular interest, as its levels are higher after open procedures [12].

The Barcelona study group acknowledges the laparoscopic advantage they identify may be attributable to it being a single center study with outcomes dependent on the surgeons’ experience. This suggests that the potential for long-term benefit from laparoscopy may be found in those who perform the procedure more frequently [13]. Although it is a single center trial, the Barcelona study has been included in meta-analyses with the three multi-institutional studies because of the large population evaluated.

## Rectal cancer

The application of laparoscopy for rectal cancer is appealing. Total mesorectal excision is the standard of care for rectal cancer and has been shown feasible laparoscopically [14-16]. The advantage of a magnified view with laparoscopy may reduce injury to the surrounding structures, including the autonomic plexus of nerves. Theoretically, this should reduce complications associated with rectal cancer resection, including urinary and sexual dysfunction.

In contrast to the available data for colon cancer, there is limited quality data for the application of laparoscopy for rectal cancer. There are several single-institution reports supporting the feasibility and equivalent long-term outcomes of laparoscopy. Nevertheless, the CLASICC study provides the highest level of evidence to date. Current on-going studies, including the American College of Surgeons Oncology Group (ACSOG) Z6051 and the Robotic versus Laparoscopic Resection for Rectal Cancer (ROLARR), aim to provide a rigorous evaluation of these approaches to the treatment of rectal cancer.

### Classic: rectal cancer

The CLASICC study found that the laparoscopic arm for rectal cancer was more likely to have undergone a total mesorectal excision. This supports the ability to maintain standard of care using a laparoscopic approach. Short-term results, however, found a trend towards positive circumferential resection margins in those undergoing laparoscopic LAR (lap: 12%, open: 6%, (p = 0.19). This raised concerns of increased risk for local recurrence [9].

Three and 5-year data, however, did not identify an increased risk for local recurrence among patients having laparoscopic operation for rectal cancer. Specifically, the 5-year local recurrence rate for LAR was 9.4% for laparoscopy and 7.6% for open (p = 0.740). Five-year data was not provided for the local recurrence rates for patients in the APR group, but there was no significant difference identified. Overall, the distant recurrence rate was 20.9% found in 111 cases at 5 years. There was no significant difference between either technique (lap: 21%, open: 20.6%; p = 0.820) [10,11].

Overall survival was also equivalent for laparoscopic and open resection of rectal cancer (lap: 60.3%, open: 52.9%; p = 0.132). Similar results were found regardless of whether patients underwent LAR or APR (LAR: lap: 62.8%, open: 56.7%;p = 0.247, APR: lap: 53.2%, open: 41.8%; p = 0.310). In fact, data at three years suggested a trend towards improved survival with laparoscopy in Dukes’ A patients, though this did not persist at 5 years (p = 0.491). This suggests that there is no difference in overall survival at 5 years between treatment arms for any stage of rectal cancer [10,11]. Cancer-free survival at 5 years was also not significantly different for patients with rectal cancer (lap: 53.2%, open: 52.1%; p = 0.953). The 5 year cancer-free survival was not significantly different for LAR (lap: 57.7%, open: 57.6%; p = 0.832) or for APR (lap: 41.4%, open: 36.2%; p = 0.618).

Patients requiring conversion from laparoscopic to open operation during resection for rectal cancer fare worse with significantly decreased 5 year overall survival (lap: 62.4%, open: 58.5%, conversion 49.6%; p = 0.005). Based on sensitivity analysis, this worse overall survival outcome was maintained even for surgeons with lower than average conversion rate, suggesting surgeon-related factors were unlikely related.

The Comparison of Open versus laparoscopic surgery for mid and low Rectal cancer After Neoadjuvant chemoradiotherapy (COREAN) trial is another randomized trial recently published showing equivalent short-term oncologic outcomes between open and laparoscopic surgery for rectal cancer. The trial included 340 patients with cT3N0-2 mid or low rectal cancer without distant metastasis. Conversion to an open procedure occurred in 2 patients (1.2%). Along with equivalence of oncologic outcomes, other short-term outcomes such as recovery of gastrointestinal function, analgesia requirement, and return to physical function showed improvement in the laparoscopic group [17].

COLOR II is an ongoing non-inferiority RCT that has 27 participating international sites with 739 patients, focusing on the outcomes from laparoscopic versus open rectal cancer resection. [18,19] Several individual studies have shown that laparoscopic total mesorectal excision is feasible and safe but CLASSICC is the only RCT to reliably support laparoscopic resection as an adequate and equivalent treatment for rectal cancer. Inclusion criteria for COLOR II include a single rectal mass within 15 cm of the anal verge on rigid proctoscopy or distal to the conjugate line on CT/MRI. Preoperative imaging is required to exclude distant metastasis. Tumors amenable to local excision and those with radiographic features suggestive of local invasion are excluded. The primary endpoint for this study will be loco-regional recurrence at 3 years.

Three other smaller, single-center RCT studies were included along with the CLASICC study in the Cochrane meta-analysis of operative approaches to rectal cancer. Those smaller trials will be discussed here. The first by Araujo et al. included 28 patients and focused on laparoscopic versus open APR after neoadjuvant therapy. No conversions were performed. Mean follow up was 47 months and only postoperative complications and local recurrence were noted. They found significantly fewer lymph nodes harvested in the laparoscopic group (5.5 nodes) compared to open (11.9) (p = 0.04). They attributed this difference to the small number of patients. At 47 months of follow up, two local recurrences were noted in the open group and none in the laparoscopic group [20].

The second trial included in the Cochrane analysis by Zhou et al. included 171 patients, comparing open and laparoscopic low and ultralow anterior resection. Included patients had the distal margin of tumor distal to the peritoneal reflection and 1.5 cm above the dentate line. Total mesenteric excision with anal sphincter preservation was accomplished in all patients. The average operative time was not significantly different (lap: 120 min, open: 106 min; p > 0.05) although blood loss was significantly less with laparoscopy (lap: 20 mL, open: 92 mL; p < 0.05). The outcomes of this study included postoperative recovery and local recurrence. There was no significant difference in days until start of fluid intake (p = 0.713) or in days of analgesia (p = 0.225). Days to first bowel movement was significantly shorter in the laparoscopic group (lap: 1.5 days, open: 2.7 days; p = 0.009). The number of hospital days was also significantly shorter in the laparoscopy group (lap: 8.1 days, open: 13.3 days; p = 0.001). Postoperative complications such as urinary retention, infection, obstruction and anastomotic leakage were significantly decreased with laparoscopy (lap: 6.1%, open: 12.4%; p = 0.016). Two port site recurrences were noted in the laparoscopic group and 3 pelvic local recurrences in the open group. Statistical significance was not reported. No mortalities were noted in the 1 to 16 month follow-up. The authors conclude that adequate resection of low rectal cancers can be performed laparoscopically but is a technically challenging approach. Long-term results regarding survival were not reported in this study [21].

The third study in the Cochrane analysis by Braga et al. followed the 5-year outcomes in 391 patients with colorectal cancer. Of this group, 134 patients had rectal cancer with 68 in the laparoscopic group and 68 in the open group. The outcomes in this study were not classified between colon and rectal cancer patients. No difference was found with respect to number of lymph nodes recovered. Distal and radial margins were negative in all patients. The rate of conversion was 4.2%. Outcomes measured included postoperative morbidity and disease-free survival. The rate of anastomotic leak was not significantly different (lap: 4.7%, open: 6.9%; p = 0.46). Reoperation was required in 6.3% of laparoscopic cases due to anastomotic leak in seven, adhesive disease in three, and bleeding in two cases compared to 9.4% of open cases, with anastomotic leak in 11, adhesive disease in six, and bleeding in two cases (p = 0.34). The length of stay was significantly shorter for the laparoscopic group (lap: 9.4 days, open: 12.7 days; p = 0.0001). Long-term complications were also noted to be significantly lower in the laparoscopic group overall (lap: 6.8%, open: 14.9%; p = 0.02). Follow-up was between 15 to 60 months. Five-year overall and disease-free survival were not significantly different between open or laparoscopic groups although p-values were not provided. Of note, the study describes that the local recurrence rate in rectal cancer was 7.3% in the laparoscopic group and 8.8% in the open group, with no p-value provided [22].

The ACSOG Z6051 study published data of their phase II pilot study in 2011 supporting that laparoscopic-assisted resection had both acceptable oncologic and perioperative clinical outcomes when compared to open resection. The pilot study was created with the intention to provide baseline parameters for a planned randomized control trial. This included the data of 54 patients with stage I to III rectal cancer obtained from 2001 to 2005. Exclusion criteria included stage IV cancer, pregnancy, and patients with ASA IV and V. Three surgeons performed the laparoscopic procedures, all with extensive laparoscopic experience (>300 procedures for colorectal disease and >20 laparoscopic rectal dissections). Pouch reconstruction and hand-assisted approaches were based on surgeon preference. Follow-up data was collected up to 5 years post-operatively.

Perioperative results described significantly earlier return of bowel function in the laparoscopic group (p = 0.03). There was no significant difference in the complication rate (lap: 22.2%, open: 32.4%; p = 0.178). Local recurrence was similar (lap: 2%, open: 4.2%, p = 0.417) as was 5-year overall survival (lap: 90.8%, open: 88.5%; p = 0.261) and disease-free survival (lap: 80.8%, open: 75.8%; p = 0.390). All cases of local recurrence occurred within 2 years. Conversion to open resection occurred in 6 cases (11.1%) and reasons included difficulty obtaining sufficient length and difficulty in completing the anastomosis. A positive circumferential rectal margin was found in one laparoscopic case, compared to 7 in the open group (p > 0.05). This was noted to be the opposite of CLASICC findings. Nevertheless the 3-year local recurrence and survival were not different between either laparoscopic or open approaches, which is also seen in the ASCOG Z6051 study.

Authors of the Z6051 pilot trial identify the study size as a major limitation, despite well-matched groups. Nevertheless, the current data suggests that the laparoscopic approach provides the potential for acceptable outcomes [23]. The Phase III randomized controlled trial arm of the study is currently recruiting patients.

As the aforementioned studies provide encouraging data supporting the efficacy of laparoscopic approaches to rectal cancer, other centers seek to evaluate the outcomes of robotic-assisted laparoscopy in this setting. Given the technical difficulties of applying laparoscopy to the confined pelvic space, robotic assistance has the advantage of providing manipulation of instruments with 7-degrees of freedom of movement, as well as enhancement of dexterity and field of view. To date, there are a number of case series as well as one published randomized trial with 18 patients showing no difference in the outcomes, conversion rates or operative time comparing robotic-assistance to standard laparoscopy [24].

The Robotic versus Laproscopic Resection for Rectal Cancer (ROLARR) trial is a prospective, randomized, controlled, multi-center, unblinded superiority which began recruiting in 2010 seeking to primarily investigate rate of conversion, circumferential margin positivity, 3-year local recurrence and overall outcomes of the robotic-assisted approach. Other outcomes of interest include cost-effectiveness and quality of life The authors aim for about 400 patients to maintain 80% power. Participating surgeons are required to have performed at least 10 robotic-assisted resections. Along with measuring overall oncologic outcomes, ROLARR seeks to evaluate the clinical benefits of robotics, including preservation of normal bladder and sexual function. Given the costs of robotic systems, these measures are essential to justify the use of robotics [25].

In conclusion, the larger CLASICC study and the three smaller single-center studies support that the laparoscopic approach to rectal cancer appears to provide an adequate oncologic excision with similar long-term outcomes. Current studies such as the Z6051, ROLARR and COLOR II studies can provide further insight on the outcomes of these minimally invasive approaches.

## Meta-analyses

Among the available research evaluating operative approach to colorectal cancer, the aforementioned studies are referred to most frequently for their prospective, multi-institutional design and large patient number. Nevertheless, each study has inherent weaknesses acknowledged by their authors. For this reason, several meta-analyses have been performed to enhance the power of the argument that laparoscopy is a safe and oncologically sound approach to the operative treatment of colorectal cancer.

The 2007 meta-analysis by Bonjer, et al. includes the four studies described above, chosen for their accrual of more than 150 patients each with data available up to 3 years post-randomization at the time of meta-analysis. Ultimately, data from 1536 patients was analyzed for outcomes in colon cancer alone. Overall results reflected the favorable outcomes from the individual studies. There was no significant difference among the studies with regard to patient age, cancer stage distribution, mean number of lymph nodes recovered (p = 0.40), rate of positive margin (p = 0.23), or postoperative mortality (p = 0.63). Three-year cancer free survival was not significantly different (p = 0.83) nor was the overall survival (p = 0.56) between laparoscopic and open resections. Tumor recurrence was noted in 121 open and 113 laparoscopic resections. The percentage of local, distant and combined local and distant recurrences was not significantly different between groups (p = 0.43, *χ*2 test). When analyzed for differences of overall survival by cancer stage, there was no significant difference identified [26].

Other meta-analyses included smaller studies in addition to the four studies analyzed by Bonjer. Despite the heterogeneity of reported results in those studies, the meta-analyses agree that there is no significant difference in survival, recurrence or lymph node recovery for open or laparoscopic resection. Jackson et al. in 2007 included both colon and rectal cases in their analysis of 10 RCTs, with the same conclusions [27].

A 2008 Cochrane Review was performed for both colon and rectal cancers, analyzing 12 trials involving 3346 patients. In colon cancer, there was no significant difference in local recurrence between treatment arms (4 RCTs with 938 patients, p = 0.57), in wound recurrence (p = 0.16) or in cancer-related mortality (5 RCTs with 1575 patients, p = 0.15). In rectal cancer, there was no significant difference in local recurrence (4 RCTs with 714 patients, p = 0.46) or cancer-related mortality (3 RCTs with 578 patients, p = 0.16) [28].

The currently available meta-analyses are reassuring for continuing laparoscopic resection for colon cancer. A meta-analysis including the current 5-year data from the larger trials would further strengthen this argument. Two of the meta-analyses also suggest that laparoscopic resection of rectal cancer may be safe and oncologically adequate, although long-term data and results of large randomized trials are forthcoming.

## Single-incision laparoscopic resection

As 5-year data supports laparoscopic resection of colon cancer with no significant increased risk of wound site recurrence, surgeons are already considering the next step in further minimizing the invasiveness of the technique. The use of SIL for colonic resection has been reported in case series, supporting its feasibility and safety [29]. Some reports include both benign and malignant disease but the small number of patients, as well as the inadequate reporting of adequate oncologic resection, limits subgroup analysis. In addition, the SIL technique adds an additional layer of technical complexity that limits widespread application at this time.

Papaconstantinou and Thomas reported a case-matched comparison in 2011 of SIL versus multiport laparoscopic colectomy for colon cancer with 1-year outcomes. The study included 26 SIL colectomy patients from 2009 to 2010 matched for factors including age, body mass index, and American Society of Anesthesiologists score. [30] Mean operative time was not significantly different at 144 minutes (p = 0.98). Total conversion rates were similar as well. The SIL group had a 12% conversion rate (2 to multiport and 1 to a hand-assisted port) and the multiport group had 15% conversion rate (3 to hand-assisted port and 1 to open). There was no difference noted regarding the number of lymph nodes harvested (SIL:18, multiport: 17; p = 0.88) or involvement of proximal, distal or radial margins (p = 0.21). The length of stay was noted to be shorter by 1.4 days in the SIL group, which was significant (p < 0.05). The mean follow-up was significantly shorter for the SIL group at 13 months compared to multiport at 21 months (p < 0.001) since all the multiport cases predated the SIL cases. At one year, there were no deaths or port-site recurrences noted. Each group was found to have 8% recurrence, all of which were distant metastasis in patients with stage III disease. One-year survival was similar at 92% for SIL and 92% for multiport (p = 0.97).

This work provides promising early evidence that the SIL may be an additional option for colon cancer resection to further reduce morbidity. The authors do not suggest SIL for rectal cancer at this time.

## Conclusions

Several large RCTs support that laparoscopic resection is not inferior to open resection of colorectal cancer. This is likely due to technical principles that are maintained during both open and laparoscopic operations, including the ligation of the primary feeding vessel at its base, minimal and atraumatic handling of the tumor, total mesorectal excision and accrual of at least 12 lymph nodes with adequate margins. Patients undergoing laparoscopic resection are in the operating room longer but are in the hospital for a shorter time. Most importantly, these key studies continue to support that an adequate oncologic resection can be performed laparoscopically without compromising outcomes.

Overall, long-term data from large RCTs support the safety and adequacy of laparoscopic resection in colon and rectal cancer. Future data from these trials will be helpful to reinforce confidence in this technique. Additional outcomes from current RCTs such as one from Australia for colon cancer and the aforementioned COLOR II trial for rectal cancer are highly anticipated. [31]. While the use of SIL as is appealing, its application and outcomes must also be evaluated as rigorously as that of laparoscopic resections for colon and rectal cancer before widespread use can be encouraged.

## Abbreviations

RCT, Randomized controlled trial; COST, Clinical Outcomes of Surgical Therapy study group; COLOR, COlon cancer Laparoscopic or Open Resection; CLASICC, Conventional versus Laparoscopic-Assisted Surgery in Colorectal Cancer; LAR, Low anterior resection; APR, Abdominoperineal resection; SIL, Single Incision Laparoscopy.

## Competing interests

The authors declare that they have no competing interests

## Authors’ contributions

All authors participated in drafting of the manuscript. All authors read and approved the final manuscript.

## References

[B1] American Cancer Society Cancer Facts and Figures 2011 2011 American Cancer Society, Atlanta, Ga

[B2] Jacobs M Verdeja JC Goldstein HS Minimally invasive colon resection (laparoscopic colectomy) Surg Laparosc Endosc 1991 1 144 150 1688289

[B3] Schwenk W Haase O Neudecker J Muller JM Short term benefits for laparoscopic colorectal resection Cochrane Database Syst Rev 2005 Jul 20 3 CD003145 10.1002/14651858.CD003145.pub2 PMC8693724 16034888

[B4] Berends FJ Kazemier G Bonjer HJ Lange JF Subcutaneous metastases after laparoscopic colectomy Lancet 1994 344 8914 58 7912321 10.1016/s0140-6736(94)91079-0

[B5] Clinical Outcomes of Surgical Therapy Study Group A comparison of laparoscopically assisted and open colectomy for colon cancer N Engl J Med 2004 350 20 2050 2059 15141043 10.1056/NEJMoa032651

[B6] Fleshman J Sargent DJ Green E Anvari M Stryker SJ Beart RW Hellinger M Flanagan R Peters W Nelson H Laparoscopic colectomy for cancer is not inferior to open surgery based on 5-year data from the COST study group trial Ann Surg 2007 246 4 655 664 17893502 10.1097/SLA.0b013e318155a762

[B7] Veldkamp R Kuhry E Hop WC Jeekel J Kazemier G Bonjer HJ Haglind E Pahlman L Cuesta MA MSika S Laparoscopic surgery versus open surgery for colon cancer: short-term outcomes of a randomized trial Lancet Oncol 2005 6 7 477 484 15992696 10.1016/S1470-2045(05)70221-7

[B8] Colon Cancer Laparoscopic or Open Resection Study Group Survival after laparoscopic surgery versus open surgery for colon cancer: long-term outcome of a randomized clinical trial Lancet Oncol 2009 10 1 44 52 19071061 10.1016/S1470-2045(08)70310-3

[B9] Guillou PJ Quirke P Thorpe H Walker J Jayne DG Smith AMH Heath RM Brown JM Short-term endpoints of conventional versus laparoscopic-assisted surgery in patients with colorectal cancer (MRC CLASICC trial): multicenter, randomized controlled trial Lancet 2005 365 9472 1718 1726 15894098 10.1016/S0140-6736(05)66545-2

[B10] Jayne DG Guillou PJ Thorpe H Quirke P Copeland J Smith AMH Heath RM Brown JM Randomized trial of laparoscopic-assisted resection of colorectal carcinoma: 3-year results of the UK MRC CLASICC trial group J Clin Oncol 2007 25 21 3061 3068 17634484 10.1200/JCO.2006.09.7758

[B11] Jayne DG Thorpe HC Copeland J Quirke P Brown JM Guillou PJ Five-year follow-up of the Medical Research Council CLASICC trial of laparoscopically assisted versus open surgery for colorectal cancer Br J Surg 2010 97 11 1638 1645 20629110 10.1002/bjs.7160

[B12] Lacy AM Garcia-Valdecasa JC Delgado S Castells A Taura P Pique JM Visa J Laparoscopy-assisted colectomy versus open colectomy for treatment of non-metastatic colon cancer: a randomized trial Lancet 2002 359 9325 2224 2229 12103285 10.1016/S0140-6736(02)09290-5

[B13] Lacy AM Delgado S Castells A Prins HA Arroyo V Ibarzabal A Pique JM The long-term results of a randomized clinical trial of laparoscopy-assisted versus open surgery for colon cancer Ann Surg 2008 248 1 1 7 18580199 10.1097/SLA.0b013e31816a9d65

[B14] Morino M Parini U Giraudo G Salval M Brachet CR Garrone C Laparoscopic total mesorectal excision: a consecutive series of 100 patients Ann Surg 2003 237 3 335 342 12616116 10.1097/01.SLA.0000055270.48242.D2 PMC1514324

[B15] Agha A Furst A Hierl J Iesalnieks I Glockzin G Anthuber M Jauch KW Schlitt HJ Laparoscopic surgery for rectal cancer: oncological results and clinical outcome of 225 patients Surg Endosc 2008 22 10 2229 2237 18622560 10.1007/s00464-008-0028-4

[B16] Leung KL Kwok SPY Lam SCW Lee JFY Yiu RYC Ng SSM Lai PBS Lau WY Laparoscopic resection of rectosigmoid carcinoma: prospective randomized trial Lancet 2004 363 1187 1192 15081650 10.1016/S0140-6736(04)15947-3

[B17] Kang SB Park JW Jeong SY Nam BH Choi HS Kim DW Lim SB Lee TG Kim DY Kim JS Chang HJ Lee HS Kim SY Jung KH Hong YS Kim JH Sohn DK Kim DH Oh JH Open versus laparoscopic surgery for mid or low rectal cancer after neoadjuvant chemoradiotherapy (COREAN trial) Lancet Oncol 2010 11 7 637 645 20610322 10.1016/S1470-2045(10)70131-5

[B18] Buunen M Bonjer HJ Hop WC Haglind E Kurlberg G Rosenberg J Lacy AM Cuesta MA D’Hoore A COLOR II Study Group COLOR II: A randomized clinical trial comparing laparoscopic and open surgery for rectal cancer Dan Med Bull 2009 56 2 89 91 19486621

[B19] Baik SH Gincherman M Mutch MG Birnbaum EH Fleshman JW Laparoscopic vs open resection for patients with rectal cancer: comparison of perioperative outcomes and long-term survival Dis Colon Rectum 2011 54 1 6 14 21160307 10.1007/DCR.0b013e3181fd19d0

[B20] Araujo SE Da Silva ESousa AH de Campos FG Habr-Gama A Dumarco RB Caravatto PP Nahas SC da Silva J Kiss DR Gama- Rodriques JJ Conventional approach x laparoscopic abdominoperineal resection for rectal cancer treatment after neoadjuvant chemoradiation: results of a prospective randomized trial Rev Hosp Clin Fac Med Sao Paulo 2003 58 3 133 140 12894309 10.1590/s0041-87812003000300002

[B21] Zhou ZG Hu M Lei WZ Yu YY Cheng Z Li L Shu Y Wang TC Laparoscopic versus open total mesorectal excision with anal sphincter preservation for low rectal cancer Surg Endosc 2004 18 1211 1215 15457380 10.1007/s00464-003-9170-1

[B22] Braga M Frasson M Vignali A Zuliani W Civelli V Di Carlo V Laparoscopic versus open colectomy in cancer patients: long-term complications, quality of life, and survival Dis Colon Rectum 2005 48 2217 2223 16228828 10.1007/s10350-005-0185-7

[B23] Baik SH Gincherman M Mutch MG Birnbaum EH Fleshman JW Laparoscopic vs open resection for patients with rectal cancer: comparison of perioperative outcomes and long-term survival Dis Colon Rectum 2011 45 6 14 21160307 10.1007/DCR.0b013e3181fd19d0

[B24] Baik SH Ko YT Kang CM Lee WJ Kim NK Sohn SK Chi HS Cho CH Robotic tumor-specific mesorectal excision of rectal cancer: short-term outcome of a pilot randomized trial Surg Endosc 2008 22 7 1601 1608 18270772 10.1007/s00464-008-9752-z

[B25] Collinson FJ Jayne DG Pigazzi A Tsang C Barrie JM Edlin R Garbett C Guillou P Holloway I Howard H Marshall H McCabe C Pavitt S Quirke P Rivers CS Brown JMB An international, multicentre, prospective, randomised, controlled, unblinded, parallel-group trial of robotic-assisted versus standard laparoscopic surgery for the curative treatment of rectal cancer Int J Colorectal Dis 2012 27 233 241 21912876 10.1007/s00384-011-1313-6

[B26] Bonjer HJ Hop WCJ Nelson H Sargent DJ Lacy AM Castells A Guillou PJ Thorpe H Brown J Delgado S Laparoscopically assisted vs open colectomy for colon cancer: a meta-analysis Arch Surg 2007 142 3 298 303 17372057 10.1001/archsurg.142.3.298

[B27] Jackson TD Kaplan GG Arena G Page JH Rogers SO Laparoscopic versus open resection for colorectal cancer: a metaanalysis of oncologic outcomes J Am Coll Surg 2007 204 3 439 446 17324779 10.1016/j.jamcollsurg.2006.12.008

[B28] Kuhry E Schwenk WF Gaupset R Romild U Bonjer HJ Long-term results of laparoscopic colorectal cancer resection Cochrane Database Syst Rev 2008 Apr 16 2 CD003432 10.1002/14651858.CD003432.pub2 PMC7017639 18425886

[B29] Champagne BJ Lee EC Leblanc F Stein SL Delaney CP Single-incision vs straight laparoscopic segmental colectomy: a case-controlled study Dis Colon Rectum 2011 54 2 183 186 21228666 10.1007/DCR.0b013e3181fd48af

[B30] Papaconstantinou HT Thomas JS Single-incision laparoscopic colectomy for cancer: assessment of oncologic resection and short-term outcomes in a case-matched comparison with standard laparoscopy Surgery 2011 150 4 820 827 22000196 10.1016/j.surg.2011.07.060

[B31] Hewett PJ Allardyce RA Bagshaw PF Frampton CM Frizelle FA Rieger NA Smith JS Solomon MJ Stephens JH Stevenson ARL Short-term outcomes of the Australasian randomized clinical study comparing laparoscopic and conventional open surgical treatments for colon cancer: the ALCCaS trial Ann Surg 2008 248 5 728 738 18948799 10.1097/SLA.0b013e31818b7595

